# Imaging of Endoplasmic Reticulum Ca^2+^ in the Intact Pituitary Gland of Transgenic Mice Expressing a Low Affinity Ca^2+^ Indicator

**DOI:** 10.3389/fendo.2020.615777

**Published:** 2021-02-16

**Authors:** Jonathan Rojo-Ruiz, Paloma Navas-Navarro, Lucía Nuñez, Javier García-Sancho, María Teresa Alonso

**Affiliations:** Instituto de Biología y Genética Molecular (IBGM), Universidad de Valladolid y Consejo Superior de Investigaciones Científicas (CSIC), Valladolid, Spain

**Keywords:** genetically encoded Ca^2+^ indicator, transgenics, ER, calcium, organelle, aequorin, pituitary

## Abstract

The adenohypophysis contains five secretory cell types (somatotrophs, lactotrophs, thyrotrophs, corticotrophs, and gonadotrophs), each secreting a different hormone, and controlled by different hypothalamic releasing hormones (HRHs). Exocytic secretion is regulated by cytosolic Ca^2+^ signals ([Ca^2+^]_C_), which can be generated either by Ca^2+^ entry through the plasma membrane and/or by Ca^2+^ release from the endoplasmic reticulum (ER). In addition, Ca^2+^ entry signals can eventually be amplified by ER release *via* calcium-induced calcium release (CICR). We have investigated the contribution of ER Ca^2+^ release to the action of physiological agonists in pituitary gland. Changes of [Ca^2+^] in the ER ([Ca^2+^]_ER_) were measured with the genetically encoded low-affinity Ca^2+^ sensor GAP3 targeted to the ER. We used a transgenic mouse strain that expressed erGAP3 driven by a ubiquitous promoter. Virtually all the pituitary cells were positive for the sensor. In order to mimick the physiological environment, intact pituitary glands or acute slices from the transgenic mouse were used to image [Ca^2+^]_ER_. [Ca^2+^]_C_ was measured simultaneously with Rhod-2. Luteinizing hormone-releasing hormone (LHRH) or thyrotropin releasing hormone (TRH), two agonists known to elicit intracellular Ca^2+^ mobilization, provoked robust decreases of [Ca^2+^]_ER_ and concomitant rises of [Ca^2+^]_C_. A smaller fraction of cells responded to thyrotropin releasing hormone (TRH). By contrast, depolarization with high K^+^ triggered a rise of [Ca^2+^]_C_ without a decrease of [Ca^2+^]_ER_, indicating that the calcium-induced calcium-release (CICR) *via* ryanodine receptor amplification mechanism is not present in these cells. Our results show the potential of transgenic ER Ca^2+^ indicators as novel tools to explore intraorganellar Ca^2+^ dynamics in pituitary gland *in situ*.

## Introduction

The anterior pituitary (AP) is a complex organ that controls a broad array of physiological functions such as growth, lactation, metabolism, or stress response (1). This functional heterogeneity is conferred by the heterogeneity in cell populations, both anatomically and functionally, that includes the core of the different axes of the endocrine system. The AP contains five endocrine cell types which control the secretion of different hormones. These include: growth hormone (GH, from somatotrophs), prolactin (PRL, from lactotrophs), follicle-stimulating hormone and luteinizing hormone (FSH and LH, from gonadotrophs), thyroid stimulating hormone (TSH, from thyrotrophs), and adrenocorticotropic hormone (ACTH, from corticotrophs).

According to the classical view, each AP cell type stores one single hormone, (or two, in the case of gonadotrophs), whose secretion is specifically regulated by a particular hypothalamic releasing hormone (HRH) (2, 3). However, distinct cell subpopulations expressing more than one single hormone have been reported (4–8). These multifunctional cells can be characterized by combining calcium imaging with labeling for multiple hormones by immunofluorescence (9). In addition to the polyhormonal cells, multi-responsive cells able to display Ca^2+^ and secretory responses to more than one HRH have also been identified by some authors (4, 10, 11). The subpopulations of multifunctional cells exhibit a striking sexual dimorphism (9), with changes during sexual cycle (12), cold stress, and along lifespan (13). Multifunctional cells are also frecuently observed in pituitary human adenomas (14, 15) and its existence may provide the basis for the paradoxical secretion and transdifferentiation (4, 16–18). However, all the above studies have only been carried out in primary cultures of rat and mouse AP cells where the interactions among different cell types and with the extracellular matrix are lost.

Recent studies using single cell transcriptomics have expanded our current knowledge on the gene expression profile associated with specific cell subtypes or AP functions in mice, rats or humans (8, 19–25).

AP provides an excellent model for endocrine excitation-secretion coupling. In the last decades AP studies have provided seminal insights into the mechanisms involved in endocrine stimulus-secretion coupling and regulation by ion channels activity. Exocytic secretion is regulated by cytosolic Ca^2+^ signals ([Ca^2+^]_C_), which can be generated either by Ca^2+^ entry from the extracellular medium through the plasma membrane and/or by Ca^2+^ release from the endoplasmic reticulum (ER) (26). Ca^2+^ influx can occur as a consequence of the transient depolarization of the plasma membrane, which opens the voltage-activated Ca^2+^ channels present in AP cells. The ER is the main Ca^2+^ store in most cells, including AP cells. The resting ER Ca^2+^ concentration ([Ca^2+^]_ER_) approaches 10^-3^ M, in contrast to the resting [Ca^2+^]_C_, which is ~10^-7^ M (27). This high [Ca^2+^]_ER_ is maintained by the equilibrium between SERCA, pumping inside the ER, and passive Ca^2+^ efflux from the ER to the cytosol through non-specific leak channels and/or through specific channels, such as the inositol trisphosphate receptor channels (IP3Rs) and/or ryanodine receptors (RyRs) (28). Binding of HRH to a G-protein coupled receptor (GPCR) leads to the activation of phospholipase Cβ (PLCβ) which hydrolyses phosphatidylinositol-4, 5- bisphosphate (PIP_2_) to inositol-1, 4, 5-trisphosphate (IP3). Due to the large Ca^2+^ gradient between the ER and the cytosol, IP3 releases Ca^2+^ from the intracellular stores, and this elicits an increase in the [Ca^2+^]_C_ and secretion of the corresponding hormone.

At least 15 subtypes of Gq/11-coupled GPCRs have been described in AP cells, as well as several receptor tyrosine kinases, whose activation leads to the mobilization of intracellular Ca^2+^ in an IP3-dependent manner (29). For example, lactotrophs and thyrotrophs are primarily activated by thyrotropin-releasing hormone (TRH) and gonadotrophs by luteinizing hormone releasing hormone (LHRH, also named GnRH). Other ligands that bind Gq/11-coupled receptors include ATP, acetylcholine, angiotensin, endothelin, serotonin, substance P, or vasoactive intestinal peptide/pituitary adenylate cyclase-activating peptide (26, 29, 30). Emptying of the ER Ca^2+^ stores can trigger the subsequent opening of the store-operated Ca^2+^ entry pathway in the plasma membrane (31, 32). This is supported by the findings that ER Ca^2+^ emptying by inhibitors of the SERCA pump (33) or blockage of the store-operated Ca^2+^ channel (32), both antagonized the secretion of adrenocorticotropin. In addition, after the initial Ca^2+^ peak, some AP cell populations displayed an oscillatory Ca^2+^ pattern. For example, in mammalian gonadotrophs, the initial Ca^2+^ pulse triggered by LHRH is typically followed by a large baseline of [Ca^2+^]_C_ oscillations, which are dependent on IP3 (34, 35). In GH3 pituitary cells, emptying of the ER Ca^2+^ stores with thapsigargin produced a sustained increase of [Ca^2+^]_C_ attributable to Ca^2+^ release and activation of store-operated calcium entry. Besides, superimposed dihydropyridine-sensitive [Ca^2+^]_C_ oscillations attributable to L-channel activity are observed (31).

In addition to activation of GPCRs, ER Ca^2+^ release can be generated by amplification of a small primary Ca^2+^ influx through calcium-induced calcium release (CICR). In frog melanotrophs, it appears that spontaneous voltage-activated Ca^2+^ influx is coupled to CICR, presumably through IP3Rs (36). Blocking Ca^2+^ entry, by removing external Ca^2+^ or adding a Ca^2+^ channel blocker, for example, will also inhibit Ca^2+^ release due to passive ER Ca^2+^ depletion. Hence, monitoring exclusively cytosolic Ca^2+^ does not unequivocally allow to discriminate between the two sources of Ca^2+^. It is, therefore, necessary to make use of specific Ca^2+^ tools that unambiguously allow identification of the origin of Ca^2+^ in response to a secretagogue. This need is even more evident in the case of endocrine AP cells, where the unique combination of ion channels, excitability mechanisms and signaling pathways determines hormone secretion in a cell specific manner. It is, therefore, crucial to monitor directly ER Ca^2+^ dynamics to unveil the unique and diverse Ca^2+^ signaling mechanisms underlying anterior pituitary cell-specific regulation.

We have recently described a new generation of ratiometric Ca^2+^ indicators (GAP, for GFP-Aequorin-Protein) that can be targeted to various organelles (37). GAP3 is optimized for measuring intraluminal Ca^2+^ in the ER matrix (38). Here we exploited the fact that erGAP3 transgenic mice express the ER-Ca^2+^ indicator GAP3 in pituitary gland, to study the contribution of the ER Ca^2+^ stores to the Ca^2+^ signals elicited by a distinct hypothalamic secretagogue. By simultaneously imaging cytosolic- and ER Ca^2+^ signals at the single cell level in an intact gland preparation, we compared the ER Ca^2+^ responses to different releasing hormones in a variety of AP preparations. Our study demonstrates the potential of a genetically encoded Ca^2+^ sensor expressed in transgenic mice for recording intraorganellar Ca^2+^ responses in intact AP.

## Methods

### Transgenic Mice

All the procedures concerning mice were approved by the animal care committee of the University of Valladolid. The generation of erGAP3 mice was described elsewhere (38). To target GAP3 to the ER (erGAP), the calreticulin signal peptide and the KDEL ER-retention peptide, were fused in frame to the 5’- and the 3’-end of the GAP gene, respectively. erGAP3 was controlled by the CAG-GS promoter (39). Mice were housed under specific pathogen-free (SPF) conditions. Tail DNA was routinely screened by PCR using two oligonucleotides, forward and reverse primers for GAP3, 5’-GATGGCAACATCCTCGGACA-3’ and 5’-GTCCTTGCTCAGGGCTGATT-3’ (234 bp product), respectively. Lines 1 and 10 of erGAP3 mice were used. Mice were maintained in heterozygosity.

### Immunofluorescence

Male and female mice were anesthetized with ketamine (80 mg/Kg) and xylazine (10 mg/Kg) and transcardially perfused with 0.9% physiological saline followed by a solution of 4% paraformaldehyde (~20 ml) and then overnight post-fixed in the same solution at 4°C. The tissue was cryoprotected in 30% sucrose and then processed for immunohistochemistry. Glands were cryosectioned (10–15 μm thick) and stored at −80 °C until use. Slides were permeabilized in PBS containing 0.5% Triton X-100 for 1 h and blocked in PBS with 10% goat serum. Slides were incubated overnight with specific antibodies against each hormone (ACTH, FSH, TSH, PRL, LH and GH) diluted (1:1,000; 1:100; 1:2,000 1:1,000; 1:1,000; 1:1,000; respectively) in fresh blocking buffer. Antisera was a generous gift from Dr. Parlow (National Hormone & Peptide Program Harbor-UCLA Medical Center, Torrance, CA). As a secondary antibody, an anti-rabbit antibody coupled to Alexa Fluor 568 (Roche) diluted 1:500 in fresh blocking buffer was used, which was incubated for 30 min. Staining controls with secondary antibody alone elicited no fluorescent signal. Nuclei were stained with Hoechst 33342. Fluorescence images were collected on a Zeiss upright Axioplan 2 microscope, using a 63X W “C-Apochromat” objective (N.A. 1.2). The fluorescence filters used were: red fluorescence: Ex 546/12, Em LP590; green fluorescence: Ex 470DF35, Em 535DF35; blue fluorescence: Ex 390/22, Em 460/50 nm. For cell quantification, positive cells randomly chosen within various fields per section corresponding to various sections per gland were analyzed using the ImageJ software. For each hormone, the percentage of labeled cells was calculated by dividing by the total number of cells, evaluated from the labeled nuclei.

### Calcium Imaging in Dissociated AP Cells

The basic protocol was previously described elsewhere (9). Briefly, mice were euthanized by cervical dislocation and the AP glands were quickly removed and digested with trypsin (1 mg/ml, Sigma) in Minimum Essential Medium (S-MEM; Gibco) for 30 min at 37°C. Dispersed cells were plated onto coverslips previously coated with poly-L-lysine-coated (0.01 mg/ml) and cultured in Dulbecco’s modified Eagle’s medium (DMEM; Gibco) supplemented with 10% fetal bovine serum and antibiotics. Experiments were performed after 2–6 h of culture. Imaging was performed as described below for slices.

### Calcium Imaging in Pituitary Slices or Entire Gland

Mice (P7-5 months) from transgenic line erGAP3 (L1 or L10) were sacrificed by cervical dislocation. AP gland was dissected out and sliced into 350–400 µm thick sections with a Mcllwain Tissue Chopper and quickly transferred to a fine-meshed membrane filter and maintained in artificial cerebrospinal fluid (ACSF) containing: 125 mM NaCl, 2.5 mM KCl, 1 mM MgCl_2_, 26 mM NaHCO_3_, 1 mM CaCl_2_, 10 mM glucose, 1.25 mM NaH_2_PO_4_, pH 7.4, continuously bubbled with a 95% O_2_/5% CO_2_ gas mixture at 25 °C. Slices were mounted onto the stage of a Zeiss Axioplan upright microscope equipped with a 20X objective (W-achroplan, Zeiss; NA= 0.5) and a Zeiss AxioCam camera MRm (12 bit) connected through a software interface (Axiovision, Zeiss) to a Xenon fluorescent excitation source and a filter wheel. GAPs were sequentially excited at 405 and 470 nm and acquired at 518–553 nm. For simultaneous measuring of [Ca^2+^]_ER_ and [Ca^2+^]_C_, slices were incubated for 1 h at room temperature with 8–12 µM Rhod-2 AM in bubbled ACSF medium. Rhod-2 was excited at 545 nm (546/12) using a dichroic mirror FT580 and light emitted was recorded above 590 nm (LP590). Pituitary slices were sequentially excited at 405, 470 (GAP) and 540 nm (Rhod). All the experiments were performed at 22–25 °C in a custom-made chamber of 42 µl volume under constant perfusion at 3 ml/min with an ‘extracellular-like solution’ containing 145 mM NaCl, 5 mM KCl, 1 mM CaCl_2_, 1 mM MgCl_2_, 10 mM glucose, and 10 mM Na-HEPES (pH 7.4). All stimuli were diluted in this extracellular-like medium and perfused for 30 s or the time indicated. Imaging of erGAP3 in the whole pituitary gland were performed similarly. Output images were captured with the AxioVision Rel 4.6.3 (Zeiss) software and pixel-to-pixel ratio analysed with ImageJ (https://imagej.nih.gov/ij/). The erGAP3 ratio R (F470/F405) was used as an index of [Ca^2+^]_ER_, and was expressed as R/R0. F540 was an index of [Ca^2+^]_C_, expressed as F/F0. R0 (or F0) was computed as the mean of the ratios (or F540) obtained during the first five to 10 frames of each experiment.

### Statistical Analysis

The data were analysed using Origin 7 (OriginLab™) and excel. Results are expressed as mean ± SEM, as indicated

## Results

### Transgenic Mice Express erGAP3 in AP Cells

**Figure 1:**
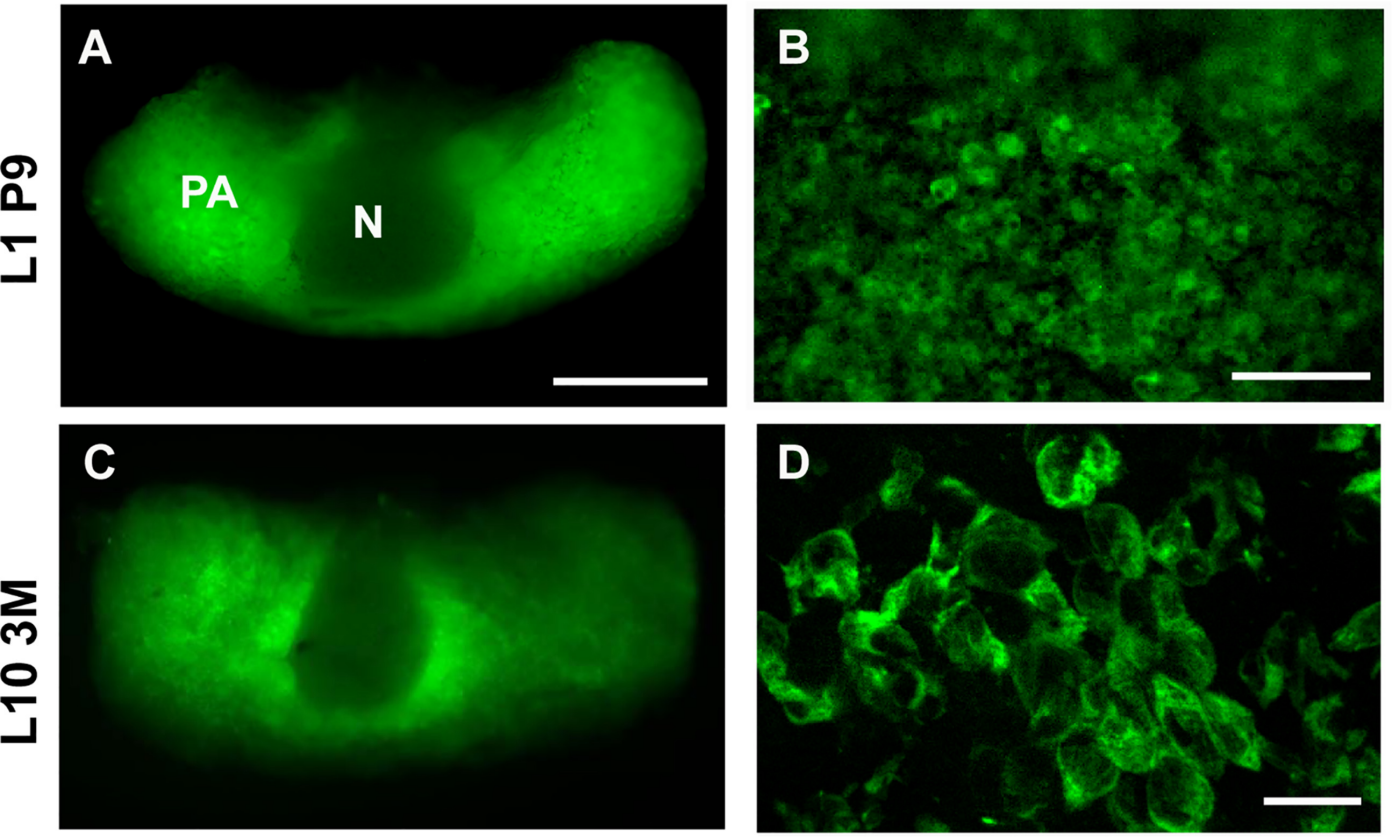
Expression of erGAP3 in the anterior pituitary (AP) of transgenic mice. **(A)** Dorsal view of an intact live pituitary isolated from a 9-day-old mouse of transgenic line L1. Image was taken at 470 nm excitation. N, neurohypophysis; PA, pars anterior. **(B)** Higher magnification view of **(A)**. **(C)** Dorsal view of intact live pituitary of a 3-months-old mouse of transgenic line L10 pituitary. **(D)** Confocal image of a cryosection (10 µm) of a fixed AP corresponding to **(C)**. Scale bar indicates 500 µm in **(A, C)**; 100 µm in **(B)**; and 20 µm in **(D)**.

In order to monitor [Ca^2+^]_ER_ in intact AP glands we used erGAP3 expressing transgenic mice in which the biosensor was controlled by the ubiquitous promoter CAG-GS (38). Using fluorescence stereomicroscopy, we easily detected the endogenous green fluorescence of erGAP3 in the pituitary gland of erGAP3 transgenic mice (**Figure 1**). The pars anterior (PA) displayed a strong green fluorescence, in contrast to the neurohypophysis (N), which was negative for erGAP. The transgene was expressed in two independent transgenic mouse lines (lines 1 and 10). Virtually all the cells were positive (mean ± SEM: 97% ± 0.2; 1,312 cells, line 10; and 788 cells, line 1). The erGAP3 fluorescence was visible both in newborn (9 days; **Figures 1A, B**) and in adult mice (3 months; **Figures 1C, D**), indicating that the transgene expression is stable along the lifespan of the mouse line.

The low Ca^2+^ affinity GAP3 (Kd~ 489 µM) was specifically targeted to the ER using the well-established strategy based on adding the signal peptide of calreticulin and the KDEL retention motif to the N-terminal and the C-terminal of the GAP gene, respectively (37, 40). The GFP positive cells showed a reticular pattern that extended throughout the entire cell and was excluded from the nucleus, as expected for localization to the endoplasmic reticulum (**Figures 1B, D**). Importantly, the GFP fluorescence was homogeneously distributed throughout the ER and no precipitates or punctate fluorescence were visible.

**Figure 2:**
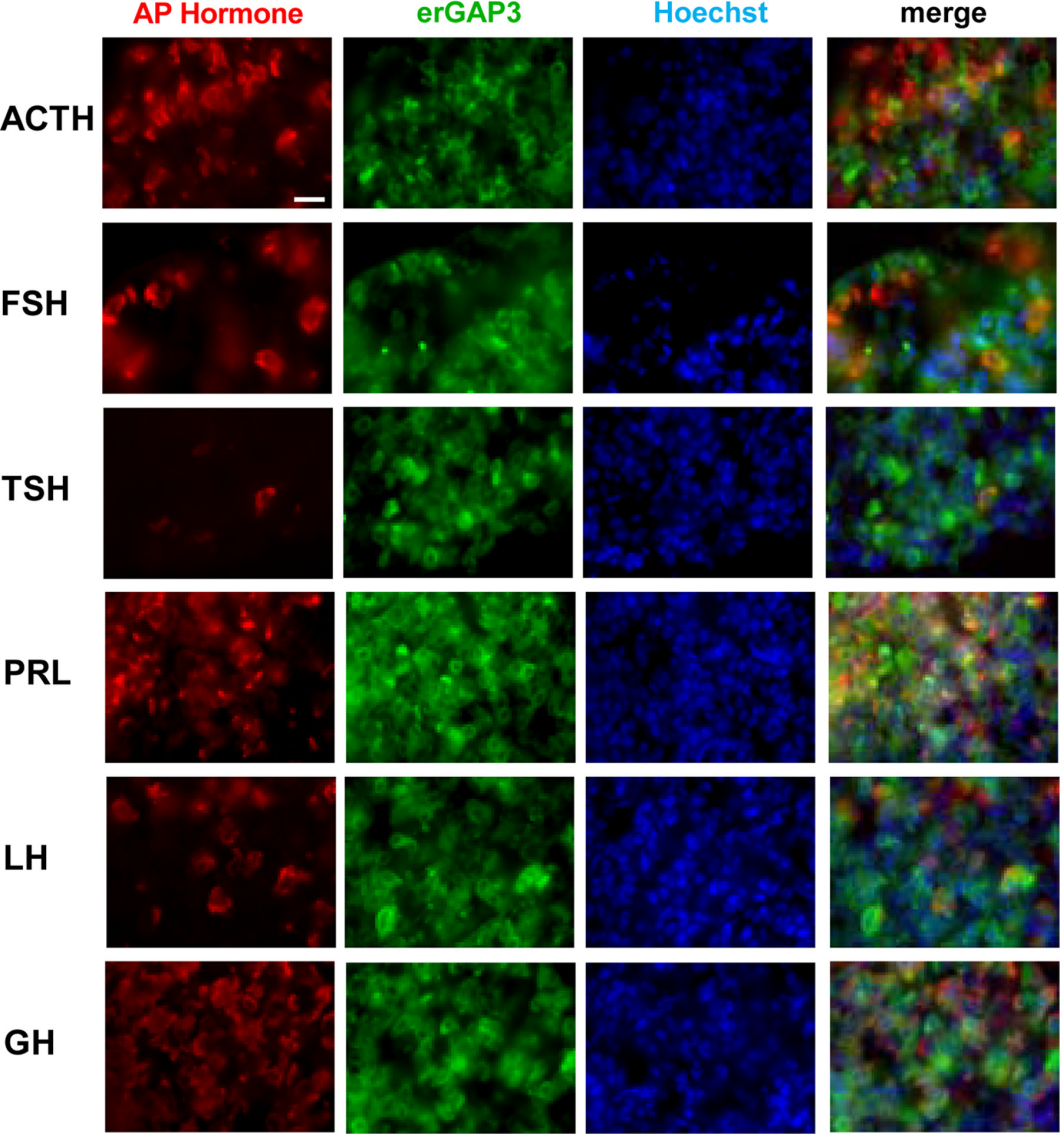
Immunohistochemical characterization of the pituitary in erGAP3 transgenic mice. An anterior pituitary (AP) cryosection was fixed and immunostained with specific antibodies against the AP hormones (ACTH, FSH, TSH, PRL, LH and GH). Nuclei were stained with Hoechst. Scale bar is 20 μm.

The cells in the AP can be classified on the basis of the stored hormone into somatotrophs (50%), lactotrophs (20%–25%), corticotrophs (10%–20%), gonadotrophs (10%), and thyrotrophs (5%) (41, 42). No apparent structural alterations were found in the pituitary gland of the erGAP3 transgenic mouse. When we examined each cell type by immunofluorescence for the presence of the stored hormones, we found that the proportions were within the expected range (**Figure 2**). Importantly, the erGAP3 indicator was expressed in all five AP cell types (between 87 and 97% for each cell type).

### erGAP3 Is Functional in Dissociated AP Cells

**Figure 3:**
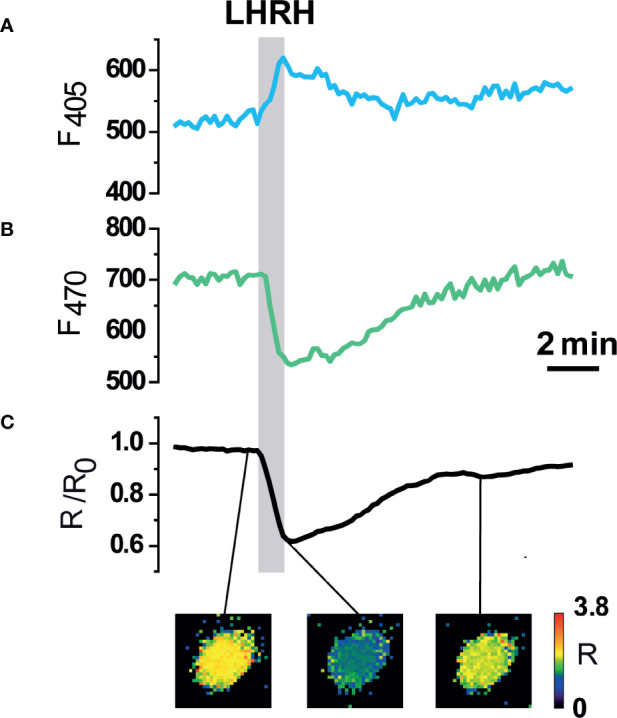
Functional expression of erGAP3 in dissociated anterior pituitary (AP) cells. **(A, B)** Individual excitation fluorescence channels (405 and 470 nm). **(C)** Ratiometric fluorescence response of erGAP3 (R) in dissociated pituitary cells isolated from transgenic line L10 (P6 mouse). Decrease of [Ca^2+^]_ER_ evoked by LHRH (100 nM). Note that the fluorescence signals at each wavelength change in the opposite direction. The decrease in the Ratio (R), expressed as R/Ro, indicates a decrease in the [Ca^2+^]_ER_. Representative trace of responses in a single cell (out of 266 erGAP3 expressing cells from 13 independent experiments from two mice) are shown. The pseudo-color images correspond to the R/R0 values at the time points in the graph (before stimuli; peak; after washing out).

The functionality of erGAP3 in pituitary cells was first examined in cultured cells dissociated from the pituitary gland of transgenic mice, since single cell imaging allows to record fluorescence changes with better optical conditions. Cultured cells displayed a robust erGAP3 expression in the ER. In the pituitary gonadotrophs, binding of LHRH to its receptor activates the Ca^2+^/inositol phosphate signaling cascade (43, 44). Cell stimulation with LHRH (100 nM) provoked the expected reciprocal fluorescent signals of the two individual GAP excitation wavelengths, with an increase of the light emission when excited at 405-nm and a decrease when excited at 470-nm that reflects the decrease of [Ca^2+^]_ER_ (**Figures 3A, B**). Calculating the ratio between the two fluorescence emission values (F470/F405) yielded a net ER Ca^2+^ decrease, a consequence of the release of Ca^2+^ from the ER into the cytosol (**Figure 3C**). This response is expected for a factor coupled to the Ca^2+^/inositol phosphate cascade. We observed a fractional decrease in the GAP3 ratio value of around 40%. The transient decrease returned to baseline levels upon agonist removal, demonstrating the reversibility of the response. In some cells, however, the ER Ca^2+^ dropped but it did not recover the initial ER Ca^2+^ level after washing. In other few cells, LHRH provoked a refilling of the ER Ca^2+^ store (not shown). Taken together, these results show that erGAP3 expression in pituitary gland displays a performance of the Ca^2+^ indicator comparable to that previously obtained in other cells types such as HeLa cells, HEK293 cells, astrocytes, or hippocampal neurones (38, 45–47).

### Simultaneous Imaging of [Ca^2+^]_ER_ and [Ca^2+^]_C_ in Acute Pituitary Slices

**Figure 4:**
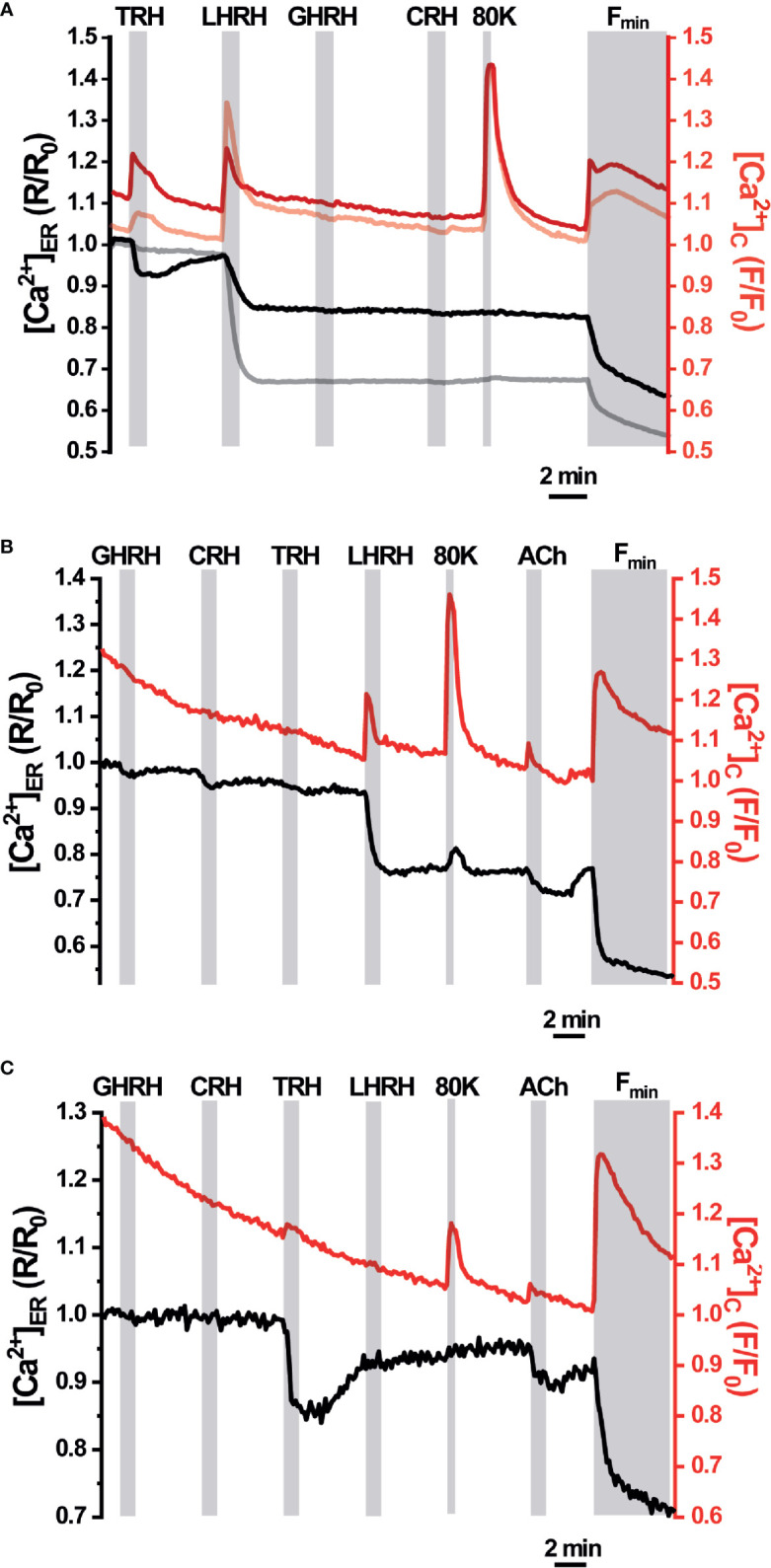
Simultaneous responses of [Ca^2+^]_ER_ and [Ca^2+^]_C_ in pituitary slices of the erGAP3 transgenic mice. Acute pituitary slices isolated from a 5-months-old erGAP3 transgenic mouse (L10) were loaded with Rhod-2. [Ca^2+^]_ER_ (black and gray traces) is represented as F470/F405 ratio (R) normalized to R_0_ (erGAP3; R/R_0_) and [Ca^2+^]_C_ represented as F/F_0_ (Rhod-2; red traces). Single cell traces are representative for at least three similar experiments. GHRH, growth hormone releasing hormone; CRH, corticotropin-releasing hormone; TRH, thyrotropin-releasing hormone; LHRH, LH releasing hormone (each hormone, 100 nM); 80K, KCl (80 mM); ACh, acetylcholine (100 μM); Fmin, depletion cocktail composed of TBH (10 μM) in Ca^2+^-free medium (EGTA 0.5 mM). **(A)** Mean traces (12 cells in the same field) that only exhibited ER Ca^2+^ changes to LHRH (grey and pale red traces). Mean traces (five cells in the same field) that exhibited ER Ca^2+^ changes to TRH and LHRH (black and intense red). **(B)** Representative traces of a LHRH-responsive cell exhibiting a small response to GHRH and to CRH. **(C)** Representative traces of a cell exclusively responsive to TRH in the same microscopic field as in B.

We next explored the ER Ca^2+^ signals in acute AP slices obtained from erGAP3 transgenic mice, where tissue structure is better preserved than in dispersed single cell cultures. Also, we recorded Ca^2+^ dynamics simultaneously in the ER and the cytosol by using erGAP3 in combination with Rhod-2, a high affinity cytosolic Ca^2+^ indicator, whose red fluorescence is spectrally compatible with that of GAP3 (**Figure 4**). The results show that most of the cells analysed (97%; 69 of 71 cells; five slices; three mice) exhibited responses to LHRH (100 nM) with a strong and rapid decrease in [Ca^2+^]_ER_ (**Figure 4A**, gray trace) and a coordinated transient in the cytosolic Ca^2+^ (**Figure 4A**, pale red trace). The addition of LHRH triggered an ER Ca^2+^ release of ~30% (the R/R0 mean ± SEM decreased from 1 down to 0.77 ± 0.01; n=69 cells) and reached a lower steady-state level. Washout of LHRH during 5 min generally failed to refill the ER, which remained half-filled after the washout. This occurred even after the stimulation of Ca^2+^ entry with a depolarizing pulse of high K^+^ (80 mM), that provoked a large cytosolic transient with no ER Ca^2+^ changes. The typical protocol finished with the perfusion of an ER depletion cocktail, used to determine the Rmin of erGAP3, that was composed of the sarco-endoplasmic reticulum Ca^2+^-ATPase (SERCA) inhibitor 2,5-ditert-butyl-benzo-hydro-quinone (TBH; 10 μM) in Ca^2+^-free medium. Interestingly, a quarter of the LHRH-responsive cells (25%; 17 of 71; five slices; three mice) also responded to thyrotropin-releasing hormone (TRH, 100 nM) with a rapid lumenal Ca^2+^ release (R/R0 (mean ± SEM) decreased from 1 down to 0.88 ± 0.05; n=18 cells; **Figure 4A**, black trace) and a coordinated small cytosolic Ca^2+^ increase (**Figure 4A**, red trace). In general, the ER Ca^2+^ drop elicited by TRH was smaller than that provoked by LHRH and, after washing out, it recovered the basal [Ca^2+^]_ER_ value observed prior to the stimulus. Moreover, the amplitude of the LHRH-induced drop was smaller in these multireceptorial cells than that observed in the LHRH-responsive monoreceptorial cells (**Figure 4A**, black and gray drops). Furthermore, most LHRH positive cells did not exhibit any response to GHRH or to CRH, although a few cells showed a minute drop in the ER Ca^2+^ not correlated with any cytosolic Ca^2+^ changes (**Figure 4B**). We also found some exceptional cells that showed a response exclusively to TRH but not to LHRH (**Figure 4C**). Addition of acetylcholine (100 µM) triggered moderate opposing Ca^2+^ transients in all cells, temporally coincident in the ER and the cytosol. It is noticeable that the depolarizing high K^+^ medium induced an increase in the cytosolic Ca^2+^ without exhibiting any significant change in the ER Ca^2+^ store. We did not find any ER Ca^2+^ release parallel to the increase in the cytosol, indicating that CICR mechanism acting through ryanodine receptors is not active in these cells.

**Figure 5:**
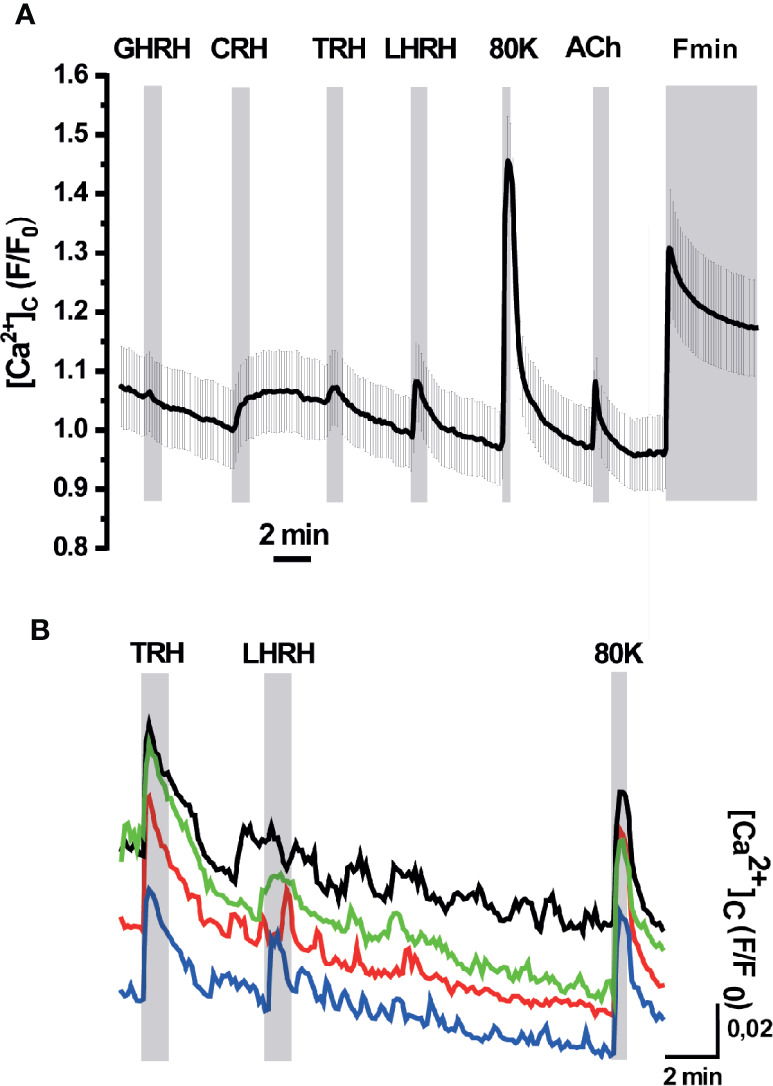
[Ca^2+^]_C_ responses in pituitary slices of erGAP3 transgenic mice. **(A)** Representative trace of [Ca^2+^]_C_ mean of the cells responsive to corticotropin-releasing hormone (CRH). Shading corresponds to SEM, n= 14 cells (out of 22 of the same experiment). Other experimental details as in **Figure 4**. **(B)** Example trace of cytosolic Ca^2+^ oscillations displayed in some cells. Individual traces have been displaced vertically for a better visibility.

Although no significant ER Ca^2+^ changes were found upon addition of the corticotropin-releasing hormone (CRH), this factor provoked a small and sustained increment in the cytosolic Ca^2+^ in a fraction of cells (F/F0 mean ± SEM; 1.03 ± 0.02; 24 of 76 cells; five slices; three mice; **Figure 5A**). This result is in agreement with the primary signaling cascade triggered by CRH, which is coupled to cAMP/PKA. Interestingly, the cells that showed a response to CRH also responded to TRH with a small but visible cytosolic Ca^2+^ transient and to LHRH with a clear spike. This result indicates the presence of multireceptorial cells. These CRH-positive cells also displayed a large Ca^2+^ spike in response to depolarization with high K^+^ and to stimulation with acetylcholine. Finally, no responses were observed to growth-hormone releasing hormone (GHRH), neither in the cytosol nor in the ER. Spontaneous or HRH-induced cytosolic Ca^2+^ oscillations, either with TRH or LHRH, were observed in some cells but no ER Ca^2+^ changes were associated with them (**Figure 5B**).

### Imaging of [Ca^2+^]_ER_ in Intact Pituitary Gland

**Figure 6:**
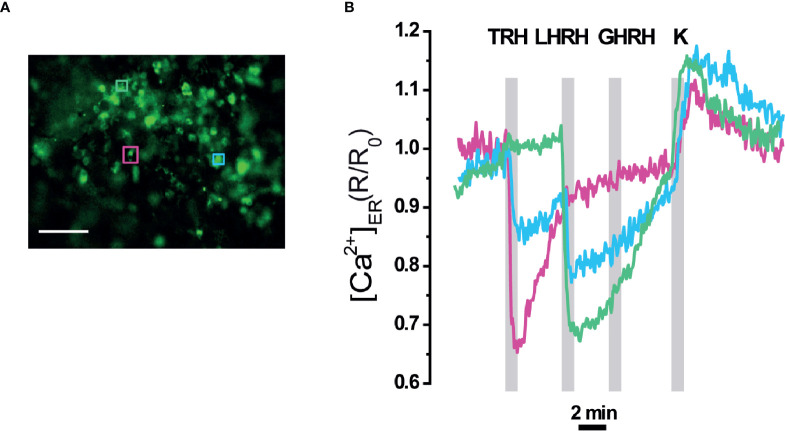
[Ca^2+^]_ER_ responses in intact pituitary gland of erGAP3 transgenic mice. **(A)** Fluorescence image (470 nm excitation) of the intact pituitary gland isolated of a P17 erGAP3 mouse. **(B)** Three different representative responses of ER Ca^2+^ corresponding to the three cells are indicated in **(A)**. Note the presence of two cells (pink and green) that each responded to only one HRH and a third cell (blue) to two hypothalamic releasing hormones (HRHs). Other experimental details as in **Figure 4**.

A higher level of tissue preservation was achieved by imaging ER Ca^2+^ signals in the whole intact pituitary gland, where erGAP3 reported changes in 80% of the cells analysed (42 of 52 cells) (**Figure 6**). Three distinct patterns of ER Ca^2+^ release were observed. First, some cells (23%; 10 out of 42) responded exclusively to TRH with a rapid and large ER Ca^2+^ drop and this decrease was reversible by washing out the stimulus, allowing the refilling of the ER. A second group of cells (57%; 24 out of 42) only responded to LHRH, but not to TRH. The amplitude of the ER Ca^2+^ release triggered by LHRH is comparable to that of TRH (~30% R/R0 change) and the ER also recovered the basal [Ca^2+^]_ER_ (average ± SEM; t= 6.7 ± 0.8 min for LHRH and t =12.3 ± 5.8 min for TRH). A third population (12%; five cells out of 42) responded to both factors, TRH and LHRH, indicating the presence of multiresponsive cells bearing several types of HRH receptors. Addition of TRH as the first stimulus released a fraction of the stored ER Ca^2+^, and the second stimulus, LHRH, released an additional fraction. Interestingly, the three types of cells responded to a high K^+^ (80 mM) depolarization stimulus with a transient increase of 0.1 ± 0.01 (R/R0 mean ± SEM; n=42 cells), as a consequence of a transient Ca^2+^ uptake into the ER, and the signal quickly returned to the resting ER levels. This last result indicates that the erGAP3 sensor was not saturated at resting ER level and no sign of CICR were observed, in agreement with the results shown in slices (**Figure 4A**).

## Discussion

Dissecting Ca^2+^ dynamics and elucidating the Ca^2+^ signals interacting between organella require specific localization of the Ca^2+^ sensors, especially in complex tissues and organs. Genetically encoded Ca^2+^ indicators (GECIs) provide a powerful tool that overcomes some of the disadvantages of the synthetic probes, such as their lack of subcellular specificity or the difficulty of loading thick tissue preparations. Transgenic mice expressing GECIs have proven to be a particularly useful technology for being minimally invasive, its ease of use and its stability and width of expression. Although the number of GECIs have dramatically increased in recent years, those optimized for high Ca^2+^ compartments such as the ER are more limited. Even less frequent is the application of low Ca^2+^ affinity indicators to transgenics, which is mostly restricted to non-mammalian organisms (48). The generation of transgenic lines expressing functional Ca^2+^ indicators can be problematic, especially in mammals. One of the drawbacks frequently encountered is the reduced sensitivity of the sensor in GECI transgenic lines in comparison with that obtained *in vitro*. Some indicator proteins displayed a punctate fluorescence, often visible as nuclear precipitates, a sign of immobile, sequestered, and non-functional indicators. One possible explanation is that many of the existing GECIs use calmodulin as Ca^2+^-sensitive motifs. Calmodulin is a highly expressed protein with a wide array of effectors, and its overexpression can be problematic and can result in embryonic lethality or in insufficient signal-to-noise ratio (SNR) (49, 50). We used here a low-Ca^2+^ affinity variant of the GAP indicators, based on the jellyfish aequorin instead of the mammalian calmodulin as the moiety providing the Ca^2+^ binding sites. This property makes the binding or the sequestration of the indicator to endogenous proteins less likely, thus avoiding possible perturbations of the signal. In the two transgenic lines generated for erGAP3, expression of the indicator in pituitary gland was robust and SNR allowed readily imaging of Ca^2+^ signals in a HRH–specific manner. The changes observed in the Rmin were close to those previously reported *in vitro* (**Figure 3**) (46). We did not observe abnormalities in the pituitary gland morphology and, importantly, no nuclear fluorescent precipitates were visible (**Figure 1**).

We show here that the erGAP3 transgenic mouse lines provide a useful and novel tool in the study of pituitary Ca^2+^ dynamics. The advantages of imaging [Ca^2+^]_ER_ using these transgenic mice are several: simple tissue preparation and imaging procedures; preservation of pituitary gland organization; and sensitive, highly efficient and simultaneous ER Ca^2+^ imaging of multiple pituitary cells. It is well known that cell to cell contact in the intact tissue is crucial to retain many of the Ca^2+^ signaling patterns (51, 52). Most of previous work on pituitary excitability was undertaken on dissociated cells in short term primary cultures or in a variety of immortalized clonal cell lines, e.g., GH3 or AtT-20 cells (53). Given the heterogeneity of the pituitary gland and the cell to cell interactions, it is advantageous using an intact preparation that preserves the spatial architecture of the original gland. A few reports have been performed in acute slices (41, 54–56). In the present study, Ca^2+^ imaging was performed both in gland slices and in whole intact gland. In both preparations we detected robust and reproducible ER Ca^2+^ signals in a HRH specific manner (**Figures 4**–**6**). The combination of cytosolic and ER Ca^2+^ measurements in pituitary slices demonstrated that the ER Ca^2+^ is the main source of the cytosolic Ca^2+^ response to TRH and LHRH, in accordance with the signaling cascade triggered by these factors (44, 57) (**Figures 4** and **5**). By contrast, ER Ca^2+^ would contribute minimally to the signals elicited by GHRH or CRH. Finally, we identified some cells that released ER Ca^2+^ in response to two secretagogues. This result was observed in the three preparations studied and confirmed the presence of multi-responsive cells bearing multiple types of receptors cells, as previously described by our and others groups (4, 10, 11). Interestingly, in a recent study using sc-transcriptome, the authors found a cell population with a unique multi-hormone gene expression profile that would reveal an unanticipated cellular complexity and plasticity in adult pituitary (8). Finally, our data indicate that ryanodine receptors are not operative in AP cells, a finding consistent with recent scRNAseq analyses of AP cells (58).

In this study, we did not focus on the exact quantification of each specific subpopulation within the gland. Instead other methods like immunohistochemistry and immunocytochemistry have been used to assess the proportions of each cell population. Probably due to limitations in the spatio-temporal resolution of our imaging equipment, our study might favor recording the LHRH-responsive cells. These cells are larger than other AP cells and displayed stronger fluorescence changes. These two factors probably led to a higher signal-to-noise ratio (SNR). In this context, the numbers of cells reported to respond to each HRH give an indication on the approximate fractions of each population but a more detailed study with a confocal or two-photon microscope would add spatial resolution required for an exact quantification. More recently, transcriptomics studies and single cell RNA-sequencing (sc-RNA) analysis of AP have proven to be excellent tools to gain insights into the expression profiles specific for each AP population (8, 19–25, 59). The combination of this powerful methodology with organellar Ca^2+^ imaging will help to correlate specific expression patterns with Ca^2+^ signaling pathways and will expand our present knowledge on the identities of AP cell types and their functions.

The identification of each of the five AP cell types during calcium imaging studies has proven to be a challenging task. In some protocols, cells are fixed and stained at the end of the calcium imaging experiment (9, 60). This method, although it has provided relevant insights into pituitary physiology, is technically challenging and it can affect the native features of the cell. More recently, an increasing number of studies have begun to exploit mouse models in which a specific cell type is genetically labeled with a fluorescent protein under the control of specific promoters. This allows the visualization of a specific cell type and its recording in real time, and does not require its posterior manipulation. One of the promoters often used is the proopiomelanocortin (POMC) promoter, that can label an ACTH population (14, 59, 60). However, some endogenous promoters might be too weak, limiting their usage, e.g., the gonadotropin-releasing hormone promoter, that formerly failed to generate a transgenic mouse for the Ca^2+^ indicator inverse pericam due to a poor SNR (61). Given the good performance reflected here by the expression of transgenic erGAP3 controlled by a ubiquitous promoter, it seems worth generating transgenic lines of erGAP3 for specific pituitary subpopulations in future studies.

## Data Availability Statement

The raw data supporting the conclusions of this article will be made available by the authors, without undue reservation.

## Ethics Statement

The animal study was reviewed and approved by Comité de Ética en Experimentación y Bienestar animal de la Universidad de Valladolid (CEEBA).

## Author Contributions

JR-R and PN-N performed the experiments and analyses and participated in preparing the manuscript figures. LN participated in the initial dispersed cultures. All the authors participated in the conception and design of the study. MA and JG-S wrote the manuscript. All authors contributed to the article and approved the submitted version.

## Funding

This work was supported by grants from the Spanish Ministerio de Economia y Competitividad (BFU2017-83066-P) and the Consejería de Educación, Junta de Castilla y León (GR175), and the Instituto de Salud Carlos III (TerCel; RD16/0011/0003).

## Conflict of Interest

The authors declare that the research was conducted in the absence of any commercial or financial relationships that could be construed as a potential conflict of interest.

## Abbreviations

ACTH, adrenocorticotrophic hormone; AP, anterior pituitary; CICR, Ca^2+^-induced Ca^2+^-release; CRH, corticotropin releasing hormone; ER, endoplasmic reticulum; FSH, follicle-stimulating hormone; GAP, GFP-Aequorin Protein; GECI, genetically encoded calcium indicator; GFP, green fluorescent protein; GH, growth hormone; GnRH, gonadotropin releasing hormone; HRH, hypothalamic releasing hormone; LH, luteinizing hormone; LHRH, luteinizing hormone releasing hormone; PRL, prolactin; SERCA, sarco-endoplasmic reticulum Ca^2+^ ATPase; SNR, signal to noise ratio; TBH, 2,5-ditert-butylbenzohydroquinone; TRH, thyrotropin-releasing hormone; TSH, thyroid stimulating hormone; [Ca^2+^]_C_, [Ca^2+^]_ER_, Ca^2+^ concentrations in cytosol or ER, respectively.
